# The OpdQ porin of *Pseudomonas aeruginosa* is regulated by environmental signals associated with cystic fibrosis including nitrate‐induced regulation involving the NarXL two‐component system

**DOI:** 10.1002/mbo3.305

**Published:** 2015-10-12

**Authors:** Randal C. Fowler, Nancy D. Hanson

**Affiliations:** ^1^ Department of Medical Microbiology and ImmunologyCenter for Research in Anti‐Infectives and BiotechnologyCreighton University School of Medicine2500 California PlazaOmahaNebraska68178

**Keywords:** Gene expression, hypoxic, immunoblotting, NarXL, nitrate, sodium chloride.

## Abstract

*Pseudomonas aeruginosa* is a versatile opportunistic pathogen that causes chronic infections in immunocompromised hosts. Multiple porins modulate outer membrane permeability under various environmental conditions. The lung environment of cystic fibrosis (CF) patients is unique with changes occurring in nutrient availability, osmolarity, and oxygen content. Although *P. aeruginosa* gene expression is modified under these conditions, little is known about how they influence porin regulation. In this study, we evaluated the regulation of the outer membrane porin OpdQ, a member of the OprD family of porins, with regard to oxygen, nitrate, and/or NaCl levels. We demonstrated using promoter::fusion clones of *P. aeruginosa* PAO1 and clinical strains collected from CF patients that OpdQ was transcriptionally repressed under low oxygen but increased in the presence of nitrate. The nitrate‐induced regulation of OpdQ was found to be dependent on the transcription factor NarL via the NarXL two‐component system. In addition, NaCl‐induced osmotic stress increased OpdQ production among most of the clinical strains evaluated. In conclusion, these data identify for the first time that specific environmental cues associated with the CF microenvironment influence porin regulation, and that the nitrate‐induced regulation of OpdQ is associated with nitrate metabolism via the NarXL two‐component system of *P. aeruginosa*.

## Introduction

Individuals with cystic fibrosis (CF) are susceptible to chronic *Pseudomonas aeruginosa* infections, which has been associated with high morbidity and mortality (Lyczak et al. 2002; Gibson et al. 2003; Folkesson et al. 2012). Within the CF airways, *P. aeruginosa* is challenged with a heterogeneous growth environment characterized by abnormal nutrient and oxygen content, chronic exposure to antibiotics, osmotic and oxidative stress, and the influx of neutrophils (Gibson et al. 2003; Worlitzsch et al. 2002; Yoon et al. 2002; O'Sullivan and Freedman 2009).

*Pseudomonas aeruginosa* infections become chronic due in part to the oxygen‐limiting and nutrient‐rich microenvironments within the airways of CF patients. This nutrient‐rich environment significantly impacts the physiology, gene expression, and overall phenotype of *P. aeruginosa* (Burns et al. 2001; Smith et al. 2006; D'Argenio et al. 2007; Palmer et al. 2007; Hoffman et al. 2010; Workentine et al. 2013). Measurement of the oxygen levels in the sputum of CF patients has confirmed the presence of hypoxic conditions (Stutts et al. 1986; Hassett et al. 2002; Worlitzsch et al. 2002). In order to survive this hypoxic environment, *P. aeruginosa* shifts from aerobic respiration to using nitrate as an alternative terminal electron for anaerobic respiration (Chen et al. 2006; Schobert and Jahn 2010). Another physiological trait of the CF lung environment noted is the concentration of NaCl. Studies have indicated increases in the NaCl concentration, whereas other studies have found no change when compared to levels in non‐CF patients (Gilljam et al. 1989; Joris et al. 1993; Knowles et al. 1997; Hull et al. 1998).

It is possible that these environmental cues within the CF airways are triggers for initial colonization events communicated through porins within the outer membrane of the *P. aeruginosa* cell. It has been proposed that most small, water‐soluble substrates transverse the outer membrane through the OprD family of porins (Hancock and Brinkman 2002; Nikaido 2003; Tamber and Hancock 2006; Tamber et al. 2006, 2007; Eren et al. 2012). This family of porins is described as substrate‐specific, facilitating the diffusion of basic amino acids, small peptides, and antibiotics (e.g., carbapenems) into the cell (Trias and Nikaido 1990a,b). The OpdQ protein (also known as PA3038 and OccK6) is a substrate‐specific porin belonging to the OprD family OpdK subfamily of porins in *P. aeruginosa* (Tamber et al. 2006; Eren et al. 2012). In the published literature, microarray studies have identified the *opdQ* gene as one of several genes differentially expressed in biofilms and associated with quorum sensing and oxidative stress (Duan et al. 2003; Schuster et al. 2003; Mashburn et al. 2005). Furthermore, other microarray studies demonstrated that oxidative stress imposed by peracetic acid increased *opdQ* gene expression (Chang et al. 2005). The major limitation of these microarray studies is that the expression data for the *opdQ* gene was not validated by qPCR and therefore should be interpreted with caution.

The limited data in the published literature and an undefined role for the OpdQ porin of *P. aeruginosa* prompted our investigation into the regulation of OpdQ. In this study, clinical strains of *P. aeruginosa* collected from patients with CF and genetically modified clones of *P. aeruginosa* strain PAO1 were used to identify and characterize the *opdQ* promoter and evaluate the transcriptional and translational response of *opdQ* to environmental cues (e.g., nitrate, NaCl, and reduced oxygen) found in the CF airways. It was hypothesized that these environmental cues found within the CF environment affect the regulation of the OpdQ porin in clinical strains of *P. aeruginosa*.

## Experimental Procedures

### Plasmids, strains, and culture conditions

The bacterial strains and plasmids used in this study are listed in Table S1. *Pseudomonas aeruginosa* strains were grown at 37°C on a rotary shaker (155 rpm) in Mueller–Hinton broth (MHB) with or without the addition of 100 mmol/L KNO_3_ (10.11 g/L), 120 mmol/L NaCl (7.01 g/L), or a combination of both. In addition, cultures were grown in ambient (~21% oxygen) or hypoxic (2% oxygen) conditions. Hypoxic conditions were controlled using a hypoxic glove chamber (Coy Lab Products, Inc., Grass Lake, MI) with the gas‐phase exchange for the hypoxic chamber set to 2% O_2_, 5% CO_2_, and 93% N_2_ using O_2_ and CO_2_ Controllers (Coy Lab Products, Inc., Grass Lake, MI). Culture media was equilibrated in the hypoxic chamber for at least 24 h before use, while bacterial strains were plated from freezer stocks onto blood agar under ambient oxygen and immediately moved to the hypoxic chamber for hypoxic growth. For plasmid maintenance, *Escherichia coli* strains were grown in the presence of ampicillin (100 *μ*g mL^−1^), gentamicin (10 *μ*g mL^−1^), or tetracycline (30 *μ*g mL^−1^), whereas *P. aeruginosa* strains were grown in the presence of carbenicillin (200 *μ*g mL^−1^), gentamicin (30 *μ*g mL^−1^), or tetracycline (64 *μ*g mL^−1^).

### 5′ RACE

The transcriptional start site of *opdQ* was mapped using the 5′ rapid amplification of cDNA ends (5′ RACE) kit (Invitrogen, Carlsbad, CA) according to the manufacturer's instructions. Briefly, cDNA was produced from 3 *μ*g of DNase‐treated RNA using Superscript^®^ II and the reverse primer opdQ 5′ RACE1 (Table S2) followed by column purification. A homopolymeric tail of cytosines was added to the 3′ end of the purified cDNA using a kit provided terminal deoxynucleotidyl transferase. PCR and nested‐PCR using primers in Table S2 amplified 5′ RACE products. The 5′ end of the *opdQ* transcript was identified by sequence analysis of gel‐purified 5′ RACE products. The nucleotide prior to the polycytosine tail in the cDNA corresponded to the transcriptional start site of *opdQ*.

### Construction of opdQ promoter::luciferase clones

**Figure 1:**
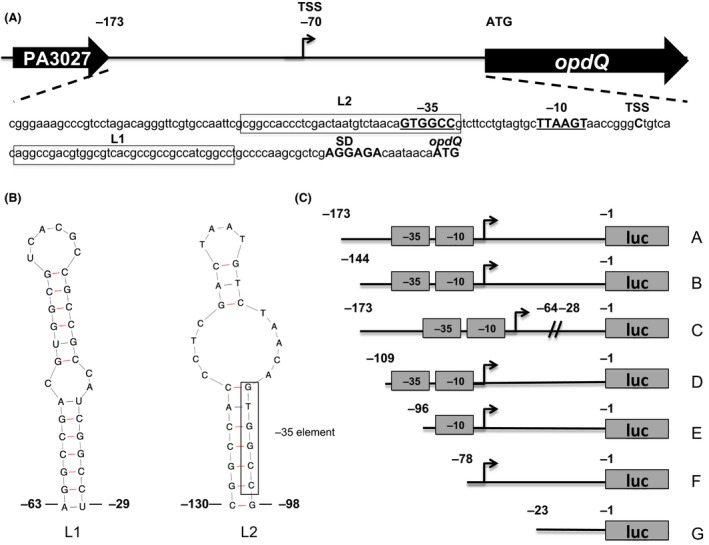
Mapping the *opdQ* promoter sequences in *Pseudomonas aeruginosa*. (A) Genetic organization of the *opdQ* structural gene and its promoter region. 5′ rapid amplification of cDNA ends and sequence analysis revealed transcription of *opdQ* in *P. aeruginosa* initiates from a single start site (TSS) at a cytosine residue 70 nucleotides upstream of the ATG of the *opdQ* structural gene. The putative ‐10 and ‐35 elements are indicated (underlined and bolded), whereas SD indicates the putative Shine–Dalgarno sequence. Boxed sequences designated L1 and L2 are nucleotides that form putative stem loops. (B) Predicted nucleic acid secondary structure of the PA3037‐*opdQ* intergenic region containing the *opdQ* promoter region showing nucleotide positions that form the L1 and L2 stem‐loop structures. These predicted structures represent the boxed sequences in (A). (C) Promoter::luciferase fusion clones representing different lengths of the *opdQ* promoter and its upstream sequences were fused to a luciferase reporter gene (*luc*) from vector pSPluc+ and subcloned into the broad‐host range vector pMP220. The *opdQ* promoter sequences (DNA fragments) and the putative ‐10 and ‐35 elements are indicated. These clones included sequences of the entire PA3037‐*opdQ* intergenic region (clone A), the removal of sequences upstream of the ‐35 element (clone B), an internal deletion of the L1 loop within the *opdQ* UTR (clone C), the removal/disruption of the L2 loop including the ‐35 element (clone D), the removal of the ‐35 element (clone E), and both ‐35 and ‐10 elements (clone F). Clone G contained only the Shine–Dalgarno sequence and served as the control/comparator in promoter activity studies.

A series of seven promoter::luciferase clones were constructed using DNA from wild‐type strain PAO1. This was achieved by fusing the putative *opdQ* promoter region of wild‐type strain PAO1 to a promoterless luciferase reporter gene (Fig. 1C). The full‐length promoter clone contained the entire *opdQ* promoter region including the putative Shine–Dalgarno sequence (SD), putative ‐10 and ‐35 region, and 67 bases of sequence upstream of the putative ‐35 element. To construct the full‐length promoter clone, primers KpnOpdQ173F and HindOpdQRBS (Table S2) that incorporated the restriction sites *Kpn*I and *Hind*III were used to amplify the entire *opdQ* promoter region and the 67 bases of sequence upstream of the putative ‐35 element using the high‐fidelity proofreading Phusion polymerase (Thermo Scientific, Rockford, IL). The PCR‐amplified product was cloned into the pJET1.2 cloning vector (Thermo Scientific) and sequenced to confirm the DNA sequence and that no mutations were introduced by PCR. The full‐length promoter construct was subcloned into the reporter gene vector pSP‐luc+ (Promega, Madison, WI) at the *Kpn*I and *Hind*III sites. The pSP‐luc+ vector containing the full‐length *opdQ* promoter region was transformed into 5‐α competent *E. coli* cells (New England BioLabs, Ipswich, MA). Following confirmation of the transformant by plasmid isolation and PCR, the full‐length clone was subcloned into the broad‐host range vector pMP220 to generate p22*luc*‐KpnOpdQ173. Once the full‐length promoter was cloned upstream of the luciferase gene in pSPluc+, additional promoter deletion clones, shorter in length than the full‐length clone, were constructed by PCR using clone A as DNA template. Subsequently, the 3′ primer XbaLucR1 was used in combination with 5′ primers listed in Table S2 to create the desired promoter deletions illustrated in Figure 1C. The PCR‐amplified promoter‐luciferase fragments were subcloned into pJET1.2, confirmed by sequencing, and further subcloned into pMP220. Alternatively, clone C was constructed by using a ultramer oligonucleotide synthesized by Integrated DNA Technologies (IDT), which included nucleotides ‐1 to ‐28 and ‐64 to ‐173 relative to the ATG of the *opdQ* within the *opdQ* promoter region (Fig. 1C). The ultramer oligonucleotide was used as DNA template with the reverse primer HindOpdQRBS to generate a 144‐bp PCR product. The same cloning strategy was used for this fragment as described above to obtain clone C within a PAO1 genetic background. Furthermore, clones A‐G in pMP220 were transformed by electroporation into PAO1. Following the creation of these promoter deletion clones, the contribution of the specific promoter sequences on gene expression was evaluated by measuring the expression of the luciferase reporter gene by qPCR.

### RNA extraction and relative quantification of transcription

Total RNA was extracted from bacterial cultures grown to mid‐log phase (OD_600_ = 0.5) using a modified TRIzol^®^ method (Invitrogen) (Wolter et al. 2009). RNA integrity was examined on a 1.5% agarose gel prior to DNase I treatment to remove contaminating DNA in the RNA samples. DNase‐treated RNA was evaluated for any remaining DNA contamination by real‐time PCR in the absence of reverse transcriptase. If little to no contaminating DNA was detected, the DNase‐treated RNA was used in subsequent experiments. qPCR was carried out according to the method described by Wolter et al. and relative quantitation of transcription was calculated using the 2^−ΔΔCT^ method (Livak and Schmittgen 2001; Wolter et al. 2009). Briefly, reactions were conducted in a one‐step reaction in 25 *μ*L volumes containing 1X Quantitect^®^ SYBR^®^ Green RT‐PCR Mastermix (Qiagen), 0.5 *μ*L of Quantitect^®^ RT mix (Qiagen, Valcencia, CA), 250 ng of DNA‐free RNA, 1X Q‐solution, and 0.5 *μ*mol/L of forward and reverse primers listed in Table S2. The *rpoD* gene was used for normalization. To ensure environmental conditions tested did not influence the transcription of the endogenous control *rpoD*, a comparison of the C_T_ values for *rpoD* using 10 clinical strains grown under the eight conditions was evaluated. This analysis showed ≤4% coefficient of variance in C_T_ values for *rpoD* among the clinical strains grown under each environmental cue. For each gene analyzed, at least three biological replicates were completed and the calculated changes in transcription were represented as fold‐changes relative to a comparator (defined for each experiment).

### Immunoblot analysis

Protein lysate from each strain was obtained from 100 mL of mid‐log phase culture grown in MHB (OD_600_ = 0.5) as described previously (Kurpiel and Hanson 2012). Fifteen micrograms of total protein from each strain was mixed with an equal volume of 2X Laemmli buffer, and denatured at 100°C for 5 min. Proteins were separated in 10% Criterion TDX Stain‐Free sodium dodecyl sulfate polyacrylamide gel electrophoresis (SDS‐PAGE) gels (Bio‐Rad, Hercules, CA). Stain‐Free gels were equilibrated in transfer buffer (25 mmol/L Tris, 192 mmol/L glycine, and 20% methanol [v/v]) for 10 min and exposed to UV light for 2.5 min to activate the Stain‐Free fluorescence, allowing the visualization of total protein. Following gel activation, proteins were electrotransferred from the gel to a methanol activated Immun‐Blot^®^ Low Fluorescent (LF) polyvinylidene difluoride (LF‐PVDF) membrane and transferred at 100 V for 1 h. After protein transfer, Stain‐Free imaging and quantification of total protein on the LF‐PVDF membrane was performed using a ChemiDoc^™^ MP imager and the ImageLab 5.1 software (Bio‐Rad). LF‐PVDF membranes were then blocked in 5% non‐fat skim milk/Tris‐buffered saline/0.1% Tween 20 (TTBS) with gentle agitation for at least 1 h. A custom polyclonal peptide antibody specific for OpdQ, directed against CKRDRETGRAQDDYG, was diluted (1:20,000) (1 mg/mL) in 5% skim milk/TTBS, and LF‐PVDF membranes were incubated with gentle agitation overnight at 4°C. The next day LF‐PVDF membranes were washed five times with TTBS for 5 min prior to incubation with horseradish peroxidase (HRP)‐goat anti‐rabbit IgG secondary antibodies (cat. no. SA1‐9510, Thermo Scientific) diluted (1:50,000) in 5% skim milk/TTBS for 1 h. LF‐PVDF membranes were washed five times with TTBS for 5 min and incubated with Supersignal West Femto maximum sensitivity detection solution (Thermo Scientific, Rockford, IL) as described by the manufacturer. The signal intensity of the chemiluminescence from the HRP‐luminol reaction was captured with a CCD camera using a ChemiDoc^™^ MP imager (Bio‐Rad). The chemiluminescent signal intensity of OpdQ then normalized to the Stain‐Free fluorescent signal intensity of total protein of reference strain PAO1 or alternative comparators (see below). If the signal intensity for OpdQ was above or below the limits of detection, the amount of protein loaded on the gel was adjusted appropriately.

### Immunoblot normalization and OpdQ quantification

**Figure 2:**
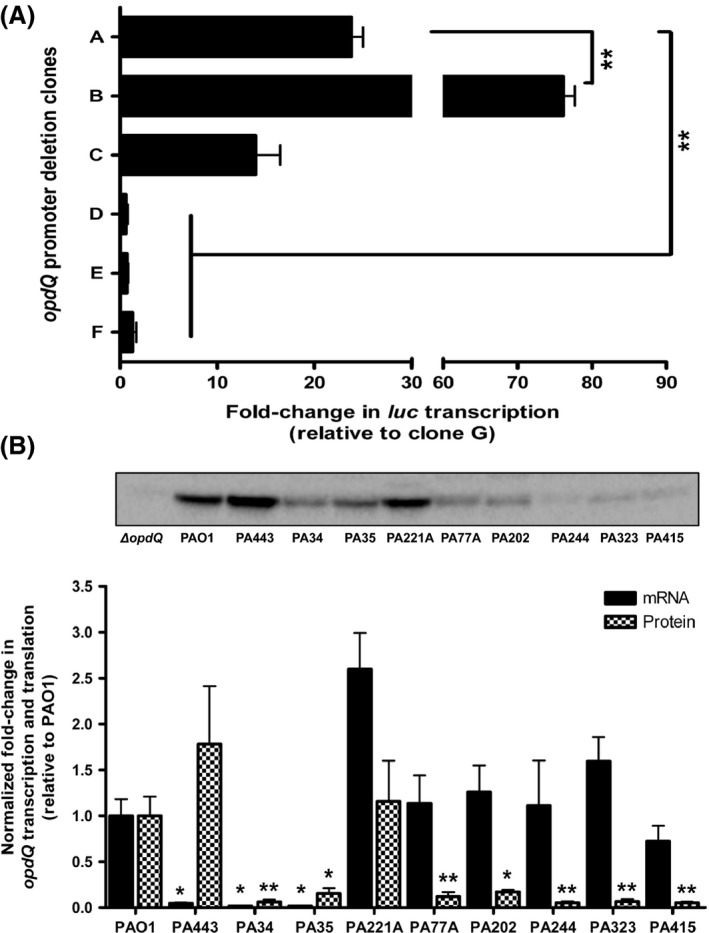
Promoter activity, transcription, and translation of OpdQ in *Pseudomonas aeruginosa* PAO1 and clinical strains grown in ambient oxygen. (A) Relative luciferase expression of *opdQ*::promoter fusion clones. Luciferase transcription was relative to clone G, normalized to *rpoD* transcription, and determined by qPCR. Clone A represents sequences of the entire PA3037‐*opdQ* intergenic region, clone B represents the removal of sequences upstream of the ‐35 element, clone C represents an internal deletion of the L1 loop within the *opdQ* UTR, clone D represents an internal deletion/disruption of the L2 loop which included the ‐35 element, clone E represents removal of the ‐35 element, and clone F represents the removal of both ‐35 and ‐10 elements. Clone G contained only the Shine–Dalgarno sequence and served as the control/comparator. Experiments were performed with at least three independent biological cultures. Error bars indicate standard deviation. Significantly different from full‐length *opdQ* promoter (clone A); **P < 0.01. (B) Transcript and protein levels of OpdQ relative to wild‐type strain PAO1. Transcription of *opdQ* in the clinical strains was compared to PAO1, normalized to *rpoD* transcription, and determined by qPCR. The amount of OpdQ protein was determined by immunoblot for PAO1, an *opdQ* knockout mutant (Δ*opdQ*), and clinical strains of *P. aeruginosa* using 15 *μ*g of whole cell lysate. Alternatively, 30 *μ*g of protein was used for strains PA34, PA35, PA77A, PA202, PA244, PA323, and PA415 that produced very low levels of OpdQ. OpdQ production was normalized to total protein by measuring Stain‐Free fluorescence on the LF polyvinylidene difluoride membrane. Three independent experiments were performed. Error bars indicate standard deviation. Significantly different fold‐changes transcription and translation of OpdQ from PAO1; *P < 0.05, **P < 0.01.

Immunoblots were normalized to total protein using Stain‐Free technology by determining the total volume intensity of each sample that was transferred to the LF‐PVDF membrane (Gurtler et al. 2013). Volume intensity refers to the Stain‐Free fluorescence of the total protein of the sample on the LF‐PVDF membrane, which is directly proportional to the total amount of protein on the LF‐PVDF membrane. The volume intensity of each protein sample was divided by the reference protein lysate (set to a value of 1) to obtain a normalization factor. The normalized chemiluminescence signal intensity was determined by multiplying the chemiluminescent signal by the normalization factor. Following normalization, the relative fold‐change in protein production was determined with respect to the reference strain. Protein lysates of PAO1 and an *opdQ* mutant were used as controls for cross‐reactivity to proteins other than OpdQ (Fig. 2B). Three biological replicates of each bacterial strain were independently collected and the average of the normalized data sets used to calculate the relative amount of OpdQ production. The linear range of detection for Stain‐Free fluorescence and the chemilumienscence signals of *P. aeruginosa* protein lysates and the anti‐OpdQ antibody was determined by performing an immunoblot on a dilution series of total protein ranging from 80 *μ*g to 0.62 *μ*g (Fig. S1). Statistical significance of OpdQ protein levels between different strains and the conditions in which they were grown were evaluated using a *t*‐test (double‐sided and paired) performed with GraphPad Prism software, version 4.0 (GraphPad Software, Inc., La Jolla, CA).

### Construction of an opdQ mutant of strain PAO1

An *opdQ* knockout mutant was constructed using the procedure described by Quenee et al. (2005). The *opdQ*‐flanking regions were amplified by PCR using Phusion polymerase and primer pairs opdQ5′EcoF and opdQ5′HindR and opdQ3′HindF and opdQ3′BamR pairs listed in Table S2. The two fragments were precipitated and then restricted with *Hind*III, column purified, coligated to generate a 158‐bp deletion within the *opdQ* gene. This fragment was cloned into the *EcoR*I–*BamH*I sites of the suicide vector pEX100Tlink. Transformants were selected on agar containing 10 *μ*g mL^−1^ gentamicin and verified by plasmid isolation and *opdQ*‐specific PCR. The *lox‐aaC1‐lox* cassette encoding gentamicin resistance was excised from pUCGmlox with *Hind*III and cloned into *Hind*III site of the *opdQ* mutant fragment. The resulting plasmid construct was transformed into the *E. coli* donor/helper strain S17.1 by electroporation followed by mobilization from *E. coli* S17.1 into *P. aeruginosa* PAO1 by conjugation. Double recombinants were selected on agar containing 5% sucrose, 1 *μ*g mL^−1^ cefotaxime, and 30 *μ*g mL^−1^ gentamicin, and confirmed by a carbenacillin (200 *μ*g mL^−1^) susceptibility and PCR screen. The removal of GmR cassette was mediated by *cre* recombinase from pCM157 followed by PCR and sequencing confirmation (Marx and Lidstrom 2002).

## Results

### Identification of the transcriptional start site and novel promoter sequences required for opdQ transcription

The first step in understanding the regulation of *opdQ* is to map its promoter region. Using 5′ RACE and sequencing, the transcriptional start site of *opdQ* was identified as a cytosine residue located 70 bp upstream of the *opdQ* translational start codon in the wild‐type *P. aeruginosa* strain PAO1 and clinical strains PA443, PA34, and PA35 (Fig. 1A). Directly upstream of the transcriptional start site were putative ‐35 (GTGGCC) and ‐10 (TTAAGT) elements that showed similarity to the ‐35 and ‐10 elements (TTGACC and TATAAT) of *σ* ^70^ in *P. aeruginosa* (Potvin et al. 2008). The predicted promoter elements of *opdQ* were located within the intergenic region between the upstream gene PA3037 and *opdQ*.

Secondary structures located within DNA or mRNA transcripts can influence transcription, mRNA stability, and/or translation of the transcript; therefore the secondary structure of the sequence located between the translation stop codon of PA3037 and the ATG of *opdQ* was evaluated using the Mfold WebServer for nucleic acids (Zuker 2003). This analysis predicted two stem loops: (1) located directly downstream of the *opdQ* transcriptional start site encompassing bases ‐63 to ‐28 and (2) located within the *opdQ* promoter region encompassing bases ‐130 to ‐98 which included the ‐35 element (Fig. 1B).

To define the DNA sequences essential for *opdQ* transcription, a series of seven *opdQ* promoter::luciferase fusion clones were constructed and luciferase transcription was measured to determine promoter activity. Figure 2A shows that luciferase transcription for clone A was 23‐fold higher than clone G, thus confirming the *opdQ* promoter was located within the PA3037‐*opdQ* intergenic region (Fig. 1A). The deletion of the nucleotides ‐173 to ‐145, immediately upstream of the ‐35 element, resulted in a 3‐fold increase in transcript levels compared to clone A, which was significantly higher than clone G (Fig. 2A). These data suggest that nucleotides ‐173 to ‐145, which include the attenuator sequence for the PA3037 transcript, slightly reduce *opdQ* transcription. To determine whether the putative stem loop in the 5′UTR of *opdQ* influenced transcription, nucleotides ‐63 to ‐29 were deleted from clone A to create clone C, representing a deletion of the L1 stem loop. Clone C exhibited a 1.7‐ and 6‐fold decrease in luciferase transcription compared to clones A and B, respectively, indicating the putative L1 stem loop plays a limited role if any in *opdQ* transcription under the conditions tested (Fig. 2A). Further evaluation of the *opdQ* promoter showed that the disruption of the putative L2 loop (clone D) through the deletion of sequences directly upstream of the ‐35 resulted in luciferase transcription similar to clone G (Fig. 2A). Subsequent deletions of the ‐35, ‐10, or both promoter elements (clones E and F) also resulted in transcript levels similar to clone G. These observations suggest that nucleotides ‐130 to ‐98 within the putative L2 stem loop may contain a *cis*‐acting element required for promoting the expression of *opdQ* (compare clones B and D in Fig. 2A).

### Discordance between transcription and translation of OpdQ among clinical strains of *P. aeruginosa*

Previous studies have primarily used laboratory‐derived strains of *P. aeruginosa* to elucidate the function of porins (Hancock and Brinkman 2002; Tamber and Hancock 2006; Tamber et al. 2007). In this study, we wanted to compare the data generated using PAO1 to data obtained using clinical strains that were collected from the host environment of the CF lung. Transcript and protein levels of OpdQ were evaluated among 10 strains of *P. aeruginosa* collected from different CF patients using the wild‐type strain PAO1 as a comparator (Table S1). The most significant differences in *opdQ* transcription ranged from a decrease of 21‐ to 58‐fold for strains PA443, PA34, and PA35 to an increase of 2.6‐fold for strain PA221A (Fig. 2B). However, strains PA77A, PA202, PA244, PA323, and PA415 expressed *opdQ* at a similar level as seen for PAO1 (black bars in Fig. 2B). To determine whether promoter mutations were influencing the transcription of *opdQ*, the *opdQ* promoter region was sequenced for all clinical strains and compared to *P. aeruginosa* PAO1. This analysis revealed that strains PA77A, PA202, PA244, PA323, and PA415 had 100% identity to *P. aeruginosa* PAO1 (accession #AE004091.2), whereas strains PA443, PA34, and PA35 had an identical base transition (A→C) and transverison (G→T) at positions ‐142 and ‐131 with respect to the translational start codon of *opdQ*. The mutations within the *opdQ* promoter region did not change the DNA structure or influence the putative formation of the stem loop structures in Figure 1B.

Protein extracts from the same cultures used to measure *opdQ* transcription were also used to evaluate the level of OpdQ protein. Strains PA443 and PA221A had equivalent levels of OpdQ compared to PAO1, whereas the other *P. aeruginosa* strains showed significant decreases in OpdQ production (checkered bars in Fig. 2B). Although OpdQ levels were comparable between PA34 and PA35, they were significantly lower than PAO1. This same trend was observed for strains PA77A, PA202, PA244, PA323, and PA415 (Fig. 2B). Surprisingly, transcript and protein levels of OpdQ varied among the clinical strains examined with strains exhibiting (1) equivalent mRNA‐to‐protein levels (e.g., PAO1, PA34, and PA35), (2) lower mRNA‐to‐protein levels (e.g., PA443), or (3) higher mRNA‐to‐protein levels for OpdQ (e.g., PA221A, PA77A, PA202, PA244, PA323, and PA415) (compare black and checkered bars, Fig. 2B). These data indicated that OpdQ is differentially expressed in clinical strains of *P. aeruginosa* and involves regulatory mechanisms that extend beyond the regulation of transcript initiation.

### Low oxygen reduces transcription and translation of OpdQ

**Figure 3:**
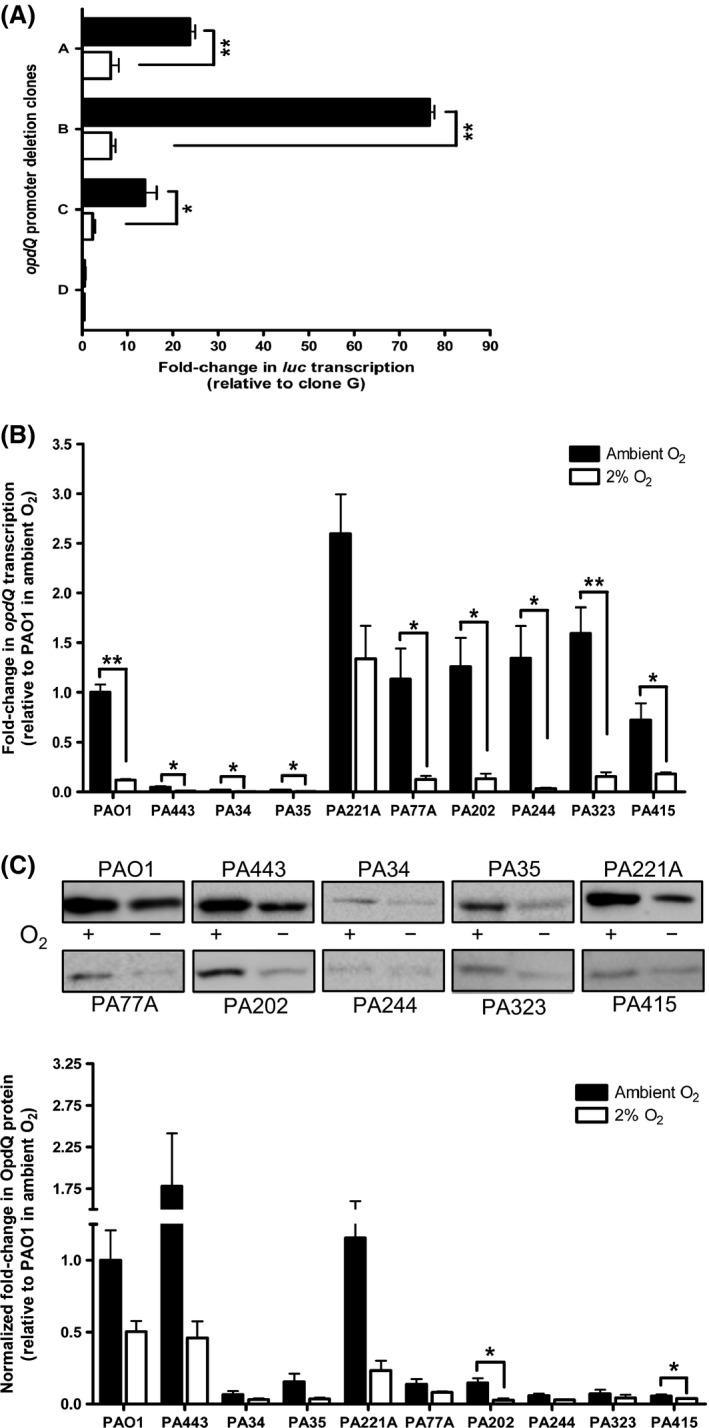
Promoter activity, transcription, and translation of OpdQ in *Pseudomonas aeruginosa* PAO1 and clinical strains. (A) Luciferase transcription levels were relative to clone G (not shown). Transcript levels (B) and protein levels (C) of OpdQ were relative to PAO1 in ambient oxygen. Transcript and protein levels of OpdQ were normalized to *rpoD* transcription and total protein, respectively. The amount of total protein used for each strain was the same as in Figure 2. Experiments were performed with at least three independent biological cultures incubated at 37°C in ambient oxygen or a hypoxic chamber (+/−) (2% O_2_, 5% CO_2_, 93% N_2_). Immunoblot images are representative of samples on the same membrane. Error bars indicate standard deviation. Significantly different from Mueller–Hinton broth cultures in ambient oxygen; *P < 0.05, **P < 0.01.

*Pseudomonas aeruginosa* infected airways of patients with CF have reduced oxygen levels (Stutts et al. 1986; Worlitzsch et al. 2002; Yoon et al. 2002; Matsui et al. 2006; Hassett et al. 2009). To assess whether low oxygen influences *opdQ* promoter activity, a hypoxic chamber was used to create a controlled low‐oxygen environment (2% O_2_, 5% CO_2_, and 93% N_2_) for bacterial growth. Promoter::fusion clones A‐G (Fig. 1C) were grown in pre‐equilibrated MHB medium within the hypoxic chamber, and luciferase transcript levels were measured. In comparison to cultures grown under ambient oxygen (~21% oxygen), promoter activity was significantly decreased for clones A–C in 2% oxygen (Fig. 3A). As observed in ambient oxygen, luciferase transcription was similar for clones D–G grown in 2% oxygen (Fig. S2). Since *opdQ* promoter activity was decreased under low oxygen compared to ambient oxygen (Fig. 3A), it was hypothesized that *opdQ* transcription would be reduced in clinical strains grown under low oxygen. Compared to expression in ambient oxygen, *opdQ* transcript levels of strains grown in 2% oxygen were significantly decreased by 5‐ to 33‐fold (Fig. 3B). Strain PA221A also showed reduced expression of *opdQ* (2‐fold), however it was not statistically significant (Fig. 3B). In general, this reduction in transcription corresponded to a 1.7‐ to 6‐fold decrease in OpdQ protein levels in the clinical strains (Fig. 3C), suggesting that in 2% oxygen OpdQ is negatively regulated at the transcriptional level.

### Nitrate enhances transcription and translation of OpdQ in low oxygen

*Pseudomonas aeruginosa* utilizes microaerophilic and nitrate metabolic pathways to grow in low oxygen (Chen et al. 2006; Alvarez‐Ortega and Harwood 2007). Since our experiments showed that the regulation of OpdQ was dependent on oxygen (Fig. 3), we wanted to determine whether nitrate affects the transcriptional and translational response of OpdQ in *P. aeruginosa*.

Experiments performed in the presence of nitrate in ambient oxygen showed that nitrate did not significantly influence promoter activity or transcript levels of *opdQ* (Fig. S3A and B). However, OpdQ protein levels increased in the presence of nitrate for a majority of the clinical strains (Fig. S3C). These data suggested that OpdQ was regulated posttranscriptionally, since significant differences in transcript levels were not observed in the presence of nitrate in ambient oxygen (Fig. S3).

**Figure 4:**
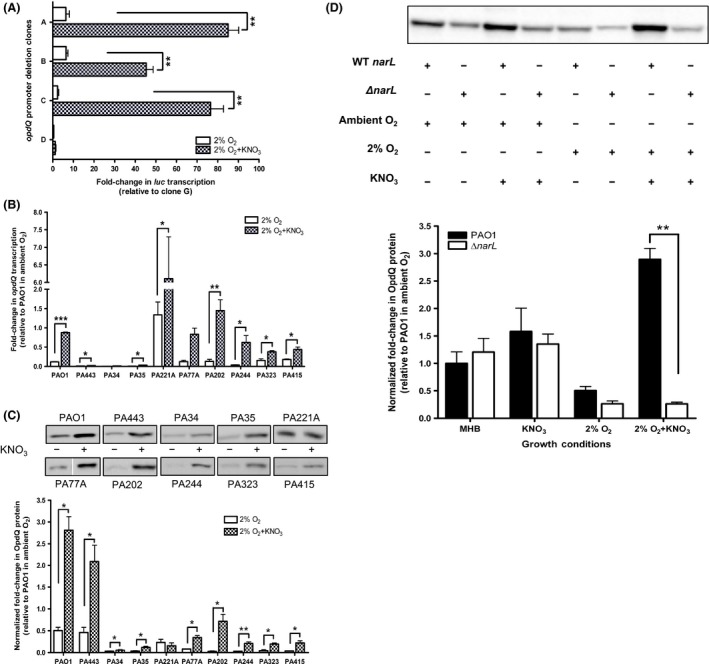
Effect of nitrate on OpdQ in clinical strains of *Pseudomonas aeruginosa*, wild‐type strain PAO1, and a Δ*narL* mutant in 2% oxygen. (A) Promoter activity was determined by measuring luciferase transcription relative to clone G (not shown) for cultures grown in 2% oxygen +/− KNO_3_. *opdQ* transcription was normalized to *rpoD* transcription (B) and the amount of OpdQ was normalized to total protein (C). The amount of total protein used for each strain was the same as in Figure 2. (D) Comparison of OpdQ levels between PAO1 (WT *narL*) and a Δ*narL* mutant +/− KNO_3_ in ambient and 2% oxygen. Experiments were performed with at least three independent biological cultures incubated at 37°C in a hypoxic chamber (2% O_2_, 5% CO_2_, and 93% N_2_). Immunoblot images are representative of samples on the same membrane. Error bars indicate standard deviation. Significantly different from Mueller–Hinton broth in 2% oxygen; *P < 0.05, **P < 0.01.

Although we saw an increase in OpdQ production in the presence of nitrate in ambient oxygen, we wanted to evaluate the effect of nitrate in 2% oxygen. Promoter activity, transcription, and protein levels of OpdQ in *P. aeruginosa* strains were grown under 2% oxygen in the presence of nitrate. The *opdQ* promoter::fusion clones were used to determine the promoter activity in the presence of nitrate in 2% oxygen. The presence of nitrate significantly increased promoter activity in clones A, B, and C (Figs. 1C, 4A) indicating that nitrate promotes *opdQ* transcription in low‐oxygen environments. To investigate this further, transcript and protein levels of OpdQ were evaluated in the wild‐type strain PAO1 and nine clinical strains. In comparison to cultures grown without nitrate in 2% oxygen, *opdQ* transcript levels for the wild‐type strain PAO1 and the clinical strains were significantly increased (7.5‐fold and 2‐ to 19‐fold, respectively) in the presence of nitrate and 2% oxygen (Fig. 4B). This upregulation in *opdQ* transcription corresponded with 5‐fold higher OpdQ protein levels for strains PAO1, PA443, PA77A, and PA323, and even higher levels of OpdQ in strains PA202, PA244, and PA415 (Fig. 4C). Strain PA221A was the only exception, showing no significant changes in OpdQ. Taken together, nitrate is able to increase the production of OpdQ in *P. aeruginosa* grown under low oxygen by activating gene expression.

### Nitrate‐induced regulation of OpdQ in low oxygen is associated with the NarXL two‐component system

*Pseudomonas aeruginosa* responds to nitrate in low oxygen via the NarXL two‐component system which functions by regulating genes involved in nitrate and nitrite respiration to control growth under hypoxic and anaerobic conditions (Alvarez‐Ortega and Harwood 2007; Schreiber et al. 2007; Schobert and Jahn 2010; He et al. 2014). To determine whether the *opdQ* promoter contains a NarL binding motif, an in silico analysis was performed. This analysis revealed two putative NarL recognition sites that match six of seven nucleotides (TGCCAAT and GACTAAT) of the published consensus sequence (TACYNMT). These binding sites were located at nucleotides ‐140 and ‐113 within the *opdQ* promoter region required for *opdQ* transcription (Fig. 1). To determine whether NarL positively regulates OpdQ in the presence of nitrate in low oxygen, the production of OpdQ was measured in a wild‐type strain PAO1 and a Δ*narL* mutant. The amount of OpdQ in the Δ*narL* mutant was similar to PAO1 regardless of nitrate being present in ambient oxygen (Fig. 4D). However, in 2% oxygen, the Δ*narL* mutant had 2‐fold lower OpdQ levels than PAO1, whereas when nitrate was present OpdQ levels were significantly lower (11‐fold; *P < *0.005) in the Δ*narL* mutant compared to PAO1 with nitrate. Taken together, these data show that NarL is associated with the nitrate‐induced regulation of OpdQ in 2% oxygen.

### Transcription and translation of OpdQ is differentially influenced by NaCl

**Figure 5:**
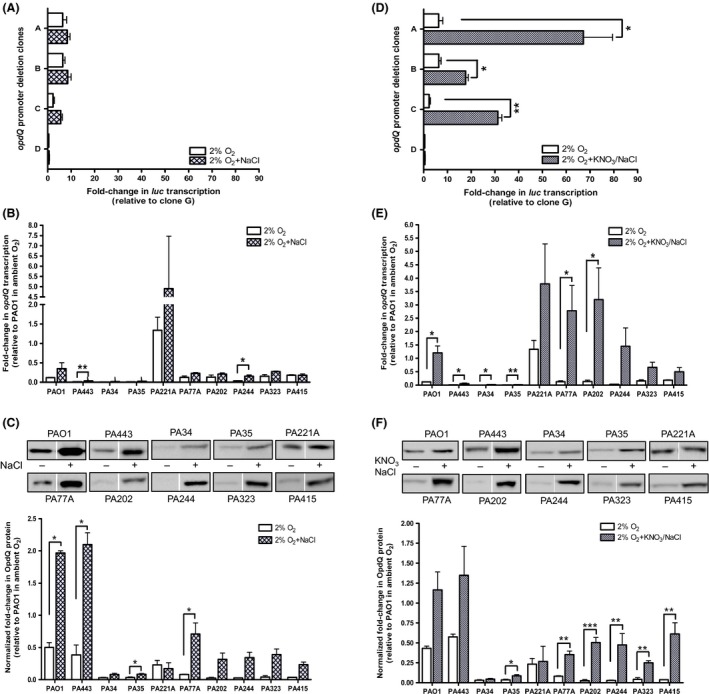
Effect of NaCl and KNO_3_/NaCl on promoter activity, transcription, and translation of OpdQ in *Pseudomonas aeruginosa* PAO1 and clinical strains grown in 2% oxygen. Luciferase transcription levels were relative to clone G (not shown) for cultures grown in 2% oxygen +/− NaCl (A and D). *opdQ* transcription was normalized to *rpoD* transcription (B and E) and the amount of OpdQ was normalized to total protein (C and F). The amount of total protein used for each strain was the same as in Figure 2. Experiments were performed with at least three independent biological cultures incubated at 37°C in a hypoxic chamber (2% O_2_, 5% CO_2_, and 93% N_2_). Immunoblot images are representative of samples on the same membrane. Error bars indicate standard deviation. Significantly different from Mueller–Hinton broth in 2% oxygen; *P < 0.05, **P < 0.01, ***P < 0.001.

Previous studies have that demonstrated high levels of NaCl are present within the CF milieu (Stutts et al. 1986; Gilljam et al. 1989; Joris et al. 1993; Gibson et al. 2003), and have been shown to impact the outer membrane through the expression of the major outer membrane porin OprF in *P. aeruginosa* (Woodruff and Hancock 1989; Brinkman et al. 1999). Since the function of OpdQ is unknown, it was postulated that the presence of NaCl and low oxygen at levels observed previously in CF patients (Gilljam et al. 1989; Joris et al. 1993; Worlitzsch et al. 2002) would influence transcription and translation of OpdQ. Promoter activity was not influenced by the presence of NaCl in 2% oxygen (Fig. 5A). This observation is further supported by a <2‐fold change in *opdQ* transcription for a majority of the clinical strains evaluated, except for the 3‐ to 5‐fold higher *opdQ* transcript levels observed for strains PA443, PA221A, and PA244 (Fig. 5B). These results are in contrast to the reduced promoter activity and transcript levels of OpdQ in *P. aeruginosa* strains grown with NaCl in ambient oxygen (Fig. S4A and B).

When the relative amount of OpdQ in each clinical strain was compared between cultures grown with and without NaCl in 2% oxygen, OpdQ protein levels increased significantly in the presence of NaCl (Fig. 5C). Figure 5C shows that the level of OpdQ in all clinical strains, positively responded to NaCl with the most significant changes observed for PAO1, PA443, PA35, and PA77A. Strains PA202, PA244, PA323, and PA415 also had increased levels of OpdQ protein that ranged from 8‐ to 13‐fold higher compared to cultures grown in 2% oxygen in the absence of NaCl (Fig. 5C). These differences were greater than the levels of OpdQ observed for PAO1, PA443, and PA77A. In summary, NaCl increased OpdQ protein levels in 2% oxygen for a majority of the strains despite the nominal changes observed in transcription (compare panels B and C, Fig. 5). This same trend was observed for seven of the 10 *P. aeruginosa* strains when NaCl was present in ambient oxygen (Fig. S4). However, not all strains (i.e., PA443, PA221A, and PA323) showed a change in OpdQ protein levels when exposed to NaCl in ambient oxygen (Fig. S4C). Taken together, these data suggest that OpdQ is differentially regulated at the transcriptional and translational levels in the presence of NaCl.

### Low oxygen, nitrate, and NaCl together effect transcription and translation of OpdQ

The lung environment of CF patients comprises a combination of high levels of NaCl (Stutts et al. 1986; Gilljam et al. 1989; Joris et al. 1993; Gibson et al. 2003), reduced oxygen (Worlitzsch et al. 2002), and increased nitrate (Hassett 1996; Linnane et al. 1998; Worlitzsch et al. 2002; Yoon et al. 2002; Palmer et al. 2007). Previous experiments performed in this study determined that nitrate and NaCl differentially influenced OpdQ individually, and when combined with low oxygen resulted in enhanced transcript and protein levels of OpdQ. To determine whether nitrate, NaCl, and low oxygen together impact the OpdQ porin of *P. aeruginosa*, the transcriptional and translational responses of OpdQ were examined. Promoter activity was significantly increased in the presence of both nitrate and NaCl in 2% oxygen (Fig. 5D). This increased promoter activity corresponded to considerably higher *opdQ* transcript levels in PAO1 and the clinical strains (Fig. 5E). The largest fold‐changes in gene expression ranged from 23‐ to 44‐fold for strains PA77A, PA202, and PA244, followed by 3‐ to 10‐fold higher *opdQ* transcript levels for strains PA34, PA35, PA323, PA443, and PAO1 relative to PAO1 in ambient oxygen. Although the fold‐change was not as pronounced in strains PA221A and PA415, they still demonstrated a 2‐ to 3‐fold increase in *opdQ* transcription relative to cultures grown in 2% oxygen without nitrate and NaCl supplementation (Fig. 5E). These results are in contrast to the reduced promoter activity and differential gene expression observed in clinical strains for the same conditions in ambient oxygen (Fig. S5A and B).

When clinical strains were evaluated under the same conditions, OpdQ protein levels increased by 2‐ to 21‐fold, except for strain PA221A. The most significant fold‐changes in OpdQ protein levels were observed for PA77A, PA202, PA244, PA323, and PA415, whereas only a 2‐ to 3‐fold increase in OpdQ production occurred for PA34, PA35, PA443, and PAO1 (Fig. 5F). Interestingly, OpdQ protein levels in the presence of nitrate and NaCl increased or did not change in ambient oxygen (Fig. S5C), despite a general reduction in gene expression and promoter activity (Fig. S5A and B). Taken together, these data indicated that OpdQ is positively regulated at the transcriptional level when subjected to nitrate and NaCl in low oxygen.

## Discussion

*Pseudomonas aeruginosa* are subjected to various environmental conditions that require quick alterations in cellular metabolism for successful colonization, growth, and survival within the confines of the CF airways. Investigators have attempted to determine the function and identify potential substrates of several porins of *P. aeruginosa* through the elucidation of protein structure and functional assays using laboratory adapted and/or derived *P. aeruginosa* strains (Tamber et al. 2006; Eren et al. 2012; Liu et al. 2012a ,b). However, in this study we sought to identify the role of the OpdQ porin through understanding its transcriptional and translational responses to environmental cues typically found within the CF airways in wild‐type and clinical strains of *P. aeruginosa*.

In this study, promoter‐mapping experiments identified a single transcriptional start site and promoter region for the *opdQ* gene in the wild‐type strain PAO1 and clinical strains of *P. aeruginosa*. Our findings suggest that nucleotides ‐110 and ‐173 within the *opdQ* promoter may contain a *cis*‐acting element that is essential for *opdQ* transcription. In addition, a palindromic sequence that forms a putative DNA cruciform was found within this essential promoter region, which included the ‐35 element (Fig. 1). The formation of DNA cruciforms due to DNA bending induced by histone‐like protein can affect transcription in prokaryotes, including *P. aeruginosa* (Perez‐Martin et al. 1994; Delic‐Attree et al. 1995).

Our findings also suggest that the *opdQ* gene is regulated at both the transcriptional and posttranscriptional levels when challenged with environmental cues found within the lung of CF patients (Fig. 2B). Kwon et al. (2014) reported similar discordant protein and mRNA levels among genes in *P. aeruginosa* strains PAO1 and PA14, attributing it to posttranscriptional mechanisms. Furthermore, transcriptome and proteomic studies have shown that steady‐state transcript abundance only partially predicts protein abundance (Vogel and Marcotte 2012). Commonly, secondary structure in the 5′ UTR of mRNA transcripts can affect the initiation of transcription, mRNA stability, and the initiation of translation in prokaryotes (Rauhut and Klug 1999; Kozak 2005; Picard et al. 2009). Upon removal of the L1 stem loop of the OpdQ promoter, transcriptional activity was reduced, (Fig. 2A) especially in 2% oxygen and in the presence of NaCl (Figs. 3A, 5A). The location of the L1 stem loop in the 5′ UTR may indicate its involvement in posttranscriptional regulation such as mRNA stability and/or translatability of the *opdQ* transcript. However, additional studies to determine the role of the L1 stem loop are required.

Oxygen availability has become the subject of intense research for trying to understand the adaptation and persistence processes of chronic *P. aeruginosa* infections in CF patients (Worlitzsch et al. 2002; Hassett et al. 2009). Transcriptome analyzes of *P. aeruginosa* have demonstrated that oxygen content affects the expression of numerous genes associated with metabolic processes, quorum sensing, biofilm formation, alginate biosynthesis, and antibiotic tolerance (Hassett 1996; Borriello et al. 2004; Alvarez‐Ortega and Harwood 2007; He et al. 2014). Our findings demonstrate that OpdQ is negatively regulated at the transcriptional level under hypoxic growth conditions (Fig. 3), which could involve quorum sensing. Previous microarray studies reported that quorum sensing molecules *N*‐3‐oxododecanoyl‐homoserine lactone and *N*‐butanoyl‐homoserine lactone decreased *opdQ* gene expression by 3.5‐fold, while *opdQ* gene expression was increased 4.4‐fold in a ∆*lasR‐rhlR* mutant (Schuster et al. 2003; Wagner et al. 2003). In several investigations, gene expression in oxygen‐limiting conditions have concluded that the LasR and RhlR quorum‐sensing systems are upregulated in hypoxic and anaerobic growth conditions (Kim et al. 2005; Alvarez‐Ortega and Harwood 2007; Duan et al. . 2003). Interestingly, two putative *las‐rhl* binding motifs with 45% identity to the *las‐rhl* box consensus and 60% identity to an atypical *las‐rhl* box were found within the L1 loop of the 5′ UTR of the *opdQ* transcript (Schuster et al. 2003; Aguirre‐Ramirez et al. 2012). These sites could serve as recognition sequences for effectors that downregulate *opdQ* transcription during hypoxic growth. It has been reported that the quorum sensing regulon is activated in low oxygen at lower cell densities (OD_660_^ ^= 0.06–0.09, ~1 × 10^8^ CFU/mL) (Pesci et al. 1997; Schuster et al. 2003). These cell densities were equivalent to the cell densities evaluated in our study (Alvarez‐Ortega and Harwood 2007). However, other mechanisms independent of quorum sensing may also regulate OpdQ since it has been shown that >200 genes were differentially expressed in 2% oxygen compared to 20% oxygen (Alvarez‐Ortega and Harwood 2007).

Recent studies have found that OpdQ among other OpdK subfamily of porins in *P. aeruginosa* showed increased specificity and selectively for negatively charged, carboxylate‐containing substrates, such as benzoate (Eren et al. 2012; Liu et al. 2012a,b). Nitrate is a negatively charged ion that is structurally similar to carboxylates (COO^−^) and restricted by the permeability of the outer membrane (Sharma et al. 2006). In this study, we demonstrated that OpdQ was responsive to nitrate posttranscriptionally in ambient oxygen and transcriptionally in 2% oxygen. It is reasonable to speculate that OpdQ may serve as a portal of entry for nitrate, particularly in low‐oxygen conditions, since its production increased as a result of nitrate availability. The repression of OpdQ observed in 2% oxygen was alleviated by the presence of nitrate, suggesting that nitrate‐responsive regulatory mechanisms are acting at the level of transcription to positively regulate OpdQ under nitrate‐rich and oxygen‐poor conditions.

During oxygen‐limiting conditions, the nitrate‐sensing NarXL two‐component system coordinates energy metabolism through activation of nitrate metabolism and the inhibition of less efficient energy generation processes such as pyruvate and arginine fermentation in *P. aeruginosa* (Benkert et al. 2008). The presence of two highly conserved NarL recognition sites within the *opdQ* region along with lack of induction of OpdQ production by nitrate in 2% oxygen in the Δ*narL* mutant (Fig. 4D), suggests that NarXL is involved in the nitrate‐responsive regulation of OpdQ in oxygen‐poor conditions. Taken together, these data suggest that OpdQ may play a role in the nitrate/nitrite respiratory pathway via the NarXL system.

In addition to nitrate, the CF airways contain higher concentrations of NaCl compared to non‐CF airways (Gilljam et al. 1989; Joris et al. 1993). Using NaCl levels previously measured in CF patients (Gilljam et al. 1989; Joris et al. 1993), we demonstrated that the transcriptional and translational response of OpdQ to NaCl varied between ambient and 2% oxygen. Although the promoter activity and transcript levels of OpdQ were mostly reduced in ambient oxygen (Fig. 3A and B), the amount of OpdQ protein was elevated in the presence of NaCl (Fig. S4C). Similarly, no significant change in promoter activity and *opdQ* transcription occurred in the presence of NaCl in 2% oxygen despite the higher OpdQ protein levels (Fig. 5C), suggesting OpdQ is regulated posttranscriptionally by mechanisms influenced by NaCl. Under osmotic stress induced by NaCl, alternative sigma factors AlgU and RpoS are activated and regulate hundreds of genes to modulate homeostasis (Potvin et al. 2008). Interestingly, many osmoprotectants contain a COO^−^ group, which are preferred substrates of the OpdK subfamily of porins, including OpdQ (Eren et al. 2012; Liu et al. 2012a,b). Taken together, these data suggest OpdQ may help maintain cellular homeostasis by trafficking solutes into the cell during osmotic stress. However, additional experiments are needed to test this hypothesis.

The combination of nitrate, NaCl, and oxygen content used in this study yielded a significant increase in promoter activity, mRNA transcription, and protein production of OpdQ (Fig. 5D and F). Although each environmental cue separately provided a different result, their combination positively impacted the regulation of OpdQ, especially in 2% oxygen. The regulatory output associated with a combination of nitrate, NaCl, and reduced oxygen content within the CF lung environment may stimulate regulatory and metabolic pathways to cope with the stress. Consequently, the individual effects of each environmental cue on the regulation of OpdQ within the CF airway may vary depending on the specific regulatory mechanism and the threshold required for activation as indicated by the fact that not every clinical strain responded in a similar manner.

In summary, our findings suggest that *P. aeruginosa* perceives and responds to specific environmental cues by altering the regulation of the OpdQ porin through unknown transcriptional and posttranscriptional regulatory mechanisms. The data presented show that the NarXL two‐component system is associated with the nitrate‐induced regulation of OpdQ in low oxygen, and that quorum sensing could be another regulatory mechanism of OpdQ. Future studies will begin to dissect the regulatory networks that govern transcription and translation of OpdQ, particularly focusing on the NarXL two‐component system and quorum sensing pathways.

## Conflict of Interest

None declared.

## Supporting information


**Figure S1.** Linear range of detection for Stain‐Free fluorescence of total protein and chemiluminescence signals of the OpdQ protein. (A) Stain‐Free image of the LF‐PVDF membrane containing a dilution series of whole cell lysate from *Pseudomonas aeruginosa* PAO1. The amount of protein loaded included – lane 1: 80 *μ*g; lane 2: 40 *μ*g; lane 3: 20 *μ*g; lane 4: 10 *μ*g; lane 5: 5 *μ*g; lane 6: 2.5 *μ*g; lane 7: 1.25 *μ*g; lane 8: 0.62 *μ*g. The Stain‐Free fluorescence was proportional to the amount of protein transferred to the LF‐PVDF membrane. Below the Stain‐Free image represents the linear regression analysis used to determine the limits of detection of the Stain‐Free signal. Linearity was achieved across 5–80 *μ*g of total protein per lane. (B) Immunoblot image of OpdQ from the same dilution series of whole lysate from *P. aeruginosa* PAO1 on the LF‐PVDF membrane in (A). The amount of protein loaded included – lane 1: 80 *μ*g; lane 2: 40 *μ*g; lane 3: 20 *μ*g; lane 4: 10 *μ*g; lane 5: 5 *μ*g; lane 6: 2.5 *μ*g; lane 7: 1.25 *μ*g; lane 8: 0.62 *μ*g. The primary antibody specific for OpdQ was diluted 1:20,000 and the goat anti‐rabbit secondary antibody was diluted 1:50,000. Chemiluminescence signal was achieved and detected using the Supersignal West Femto maximum sensitivity detection solution (Thermo Scientific, Rockford, IL) and the ChemiDoc^™^ MP imager (Bio‐Rad). Below the immunoblot in the linear regression analysis used to identify the linear range of detection for cheminluminescence signal of the OpdQ protein. Linearity was achieved across 5 to 80 *μ*g of total protein per lane. Data represent the mean SD of the chemiluminescence signals (*n* = 3). Experiments were performed with three independent immunoblots representing three independent protein lysates extracted from PAO1. Data represent the mean ± standard deviation of the Stain‐Free and chemiluminescence signals (*n* = 3).
**Figure S2.** Luciferase transcription of clone G with and without KNO_3_ and NaCl in ambient and 2% oxygen. Luciferase transcription levels were normalized to *rpoD* and relative to clone G in ambient oxygen. Experiments were performed with at least three independent biological cultures. Error bars indicate standard deviation.
**Figure S3.** Promoter activity, transcription, and translation of OpdQ in *Pseudomonas aeruginosa* PAO1 and clinical strains grown with and without KNO_3_ in ambient oxygen. (A) Luciferase transcription levels were relative to clone G (not shown). Transcript levels (B) and protein levels (C) of OpdQ were relative to PAO1 in ambient oxygen. Transcript and protein levels of OpdQ were normalized to *rpoD* transcription and total protein, respectively. The amount of total protein used for each strain was the same as in Figure 2. Experiments were performed with at least three independent biological cultures incubated at 37°C in ambient oxygen. Immunoblot images are representative of samples on the same membrane. Error bars indicate standard deviation. Significantly different from MHB cultures in ambient oxygen; *P < 0.05.
**Figure S4.** Promoter activity, transcription, and translation of OpdQ in *Pseudomonas aeruginosa* PAO1 and clinical strains grown with and without NaCl in ambient oxygen. (A) Luciferase transcription was relative to clone G (not shown). Transcript levels (B) and protein levels (C) of OpdQ were relative to PAO1 in ambient oxygen. Transcript and protein levels of OpdQ were normalized to *rpoD* transcription and total protein, respectively. The amount of total protein used for each strain was the same as in Figure 2. Experiments were performed with at least three independent biological cultures incubated at 37°C in ambient oxygen. Immunoblot images are representative of samples on the same membrane. Error bars indicate standard deviation. Significantly different from MHB cultures in ambient oxygen; *P < 0.05, **P < 0.01, ***P < 0.001.
**Figure S5.** Promoter activity, transcription, and translation of OpdQ in *Pseudomonas aeruginosa* PAO1 and clinical strains with and without KNO_3_ and NaCl in MHB medium in ambient oxygen. (A) Luciferase transcription was relative to clone G (not shown). Transcript levels (B) and protein levels (C) of OpdQ were relative to PAO1 in ambient oxygen. Transcript and protein levels of OpdQ were normalized to *rpoD* transcription and total protein, respectively. The amount of total protein used for each strain was the same as in Figure 2. Experiments were performed with at least three independent biological cultures incubated at 37°C in ambient oxygen. Immunoblot images are representative of samples on the same membrane. Error bars indicate standard deviation. Significantly different from MHB cultures in ambient oxygen; *P < 0.05, **P < 0.01.

**Table S1.** Bacterial strains and plasmids used in this study.

**Table S2.** Primers used in this study.
