# Supplementary figures and images for: The OpdQ porin of Pseudomonas aeruginosa is regulated by environmental signals associated with cystic fibrosis including nitrate‐induced regulation involving the NarXL two‐component system
Source: Microbiologyopen. 2015 Oct 12;4(6):967–82. doi: 10.1002/mbo3.305 (PMC4694141; doi:10.1002/mbo3.305)

A

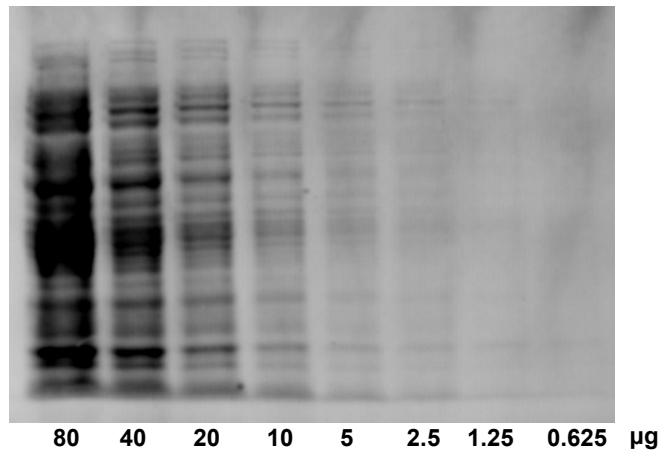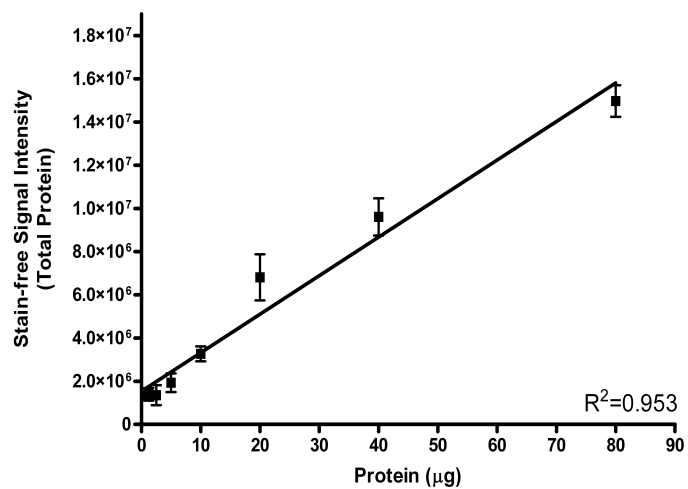

B

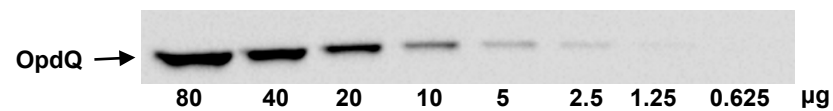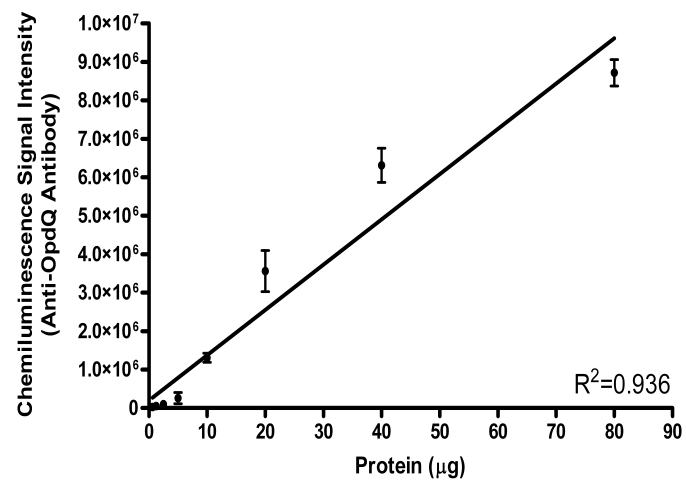

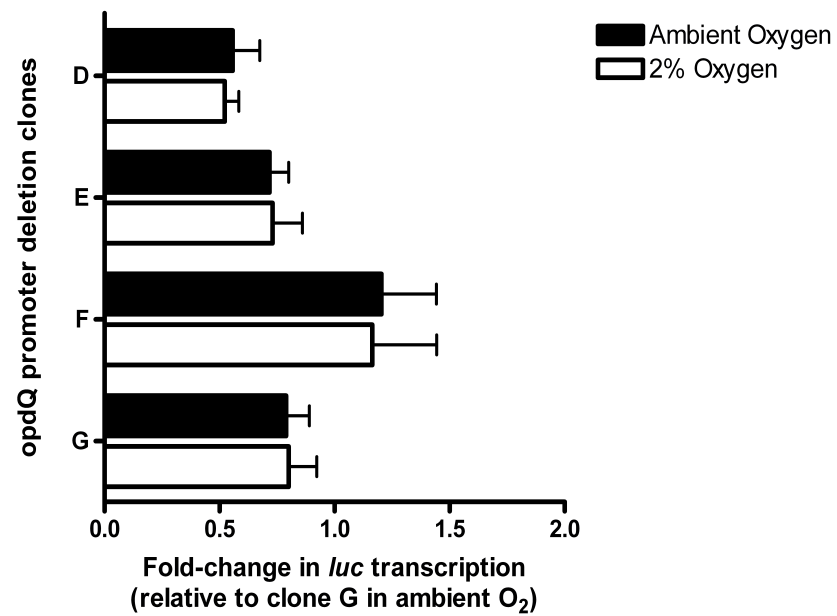

# Ambient O<sub>2</sub>

**A**

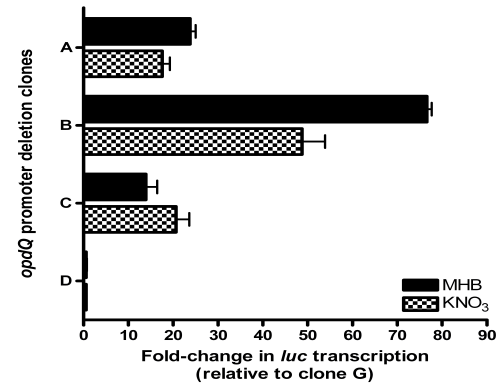

**B**

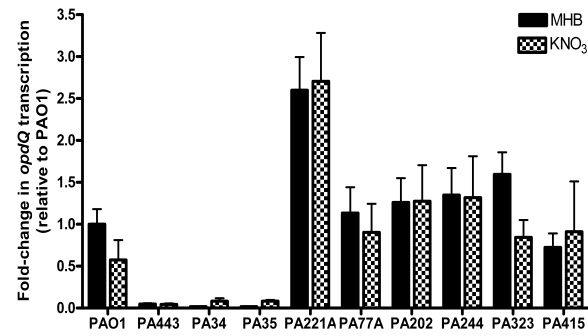

**C**

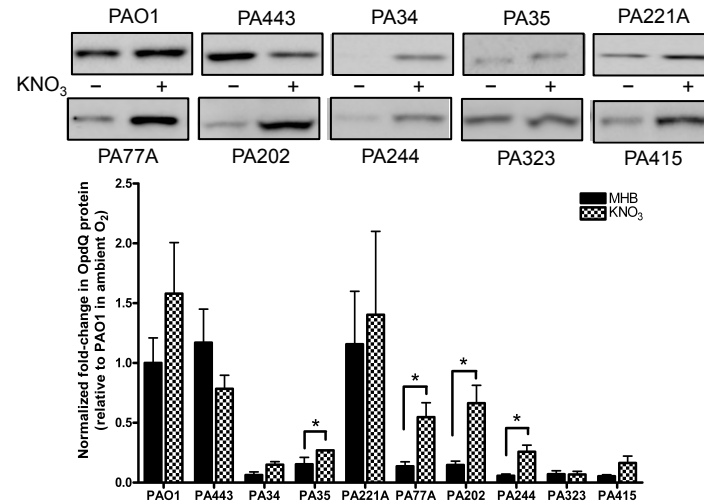

# Ambient O<sub>2</sub>

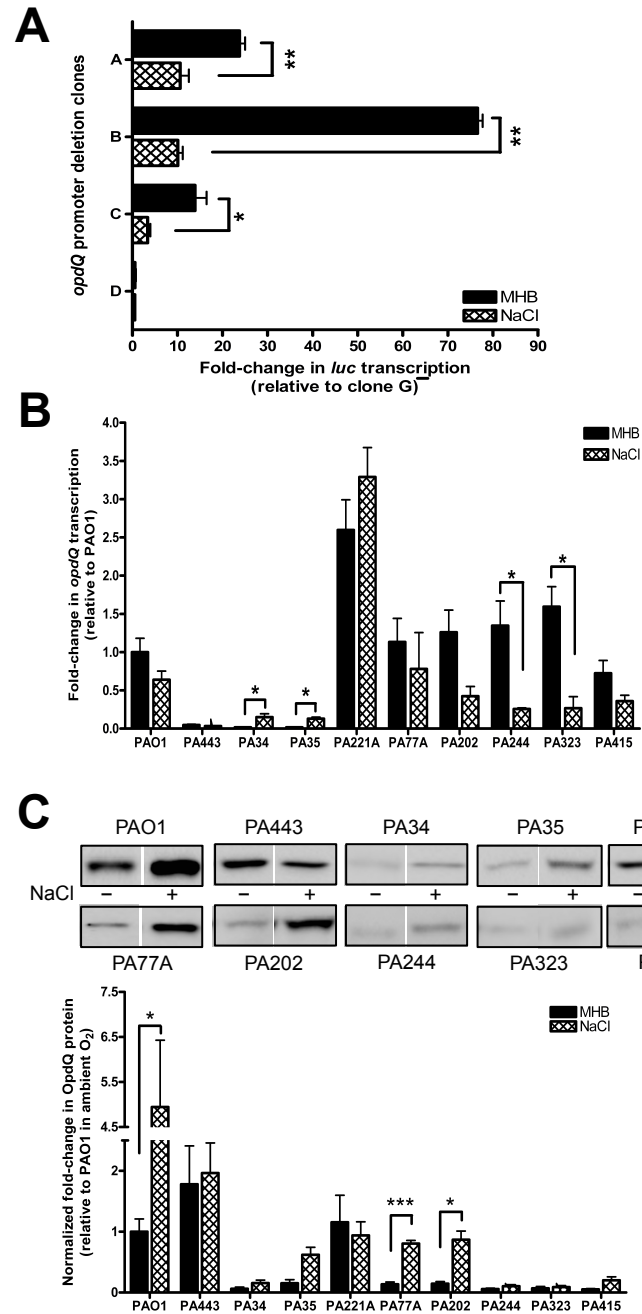

# Ambient O<sub>2</sub>

**A**

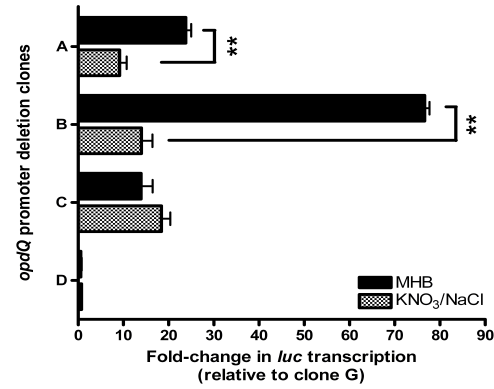

**B**

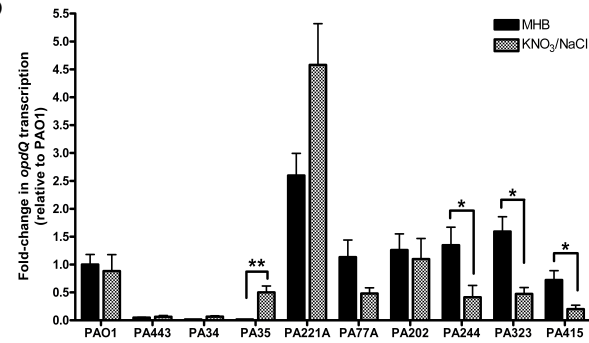

**C**

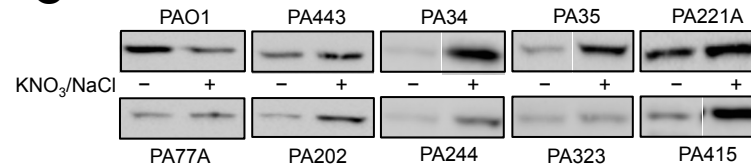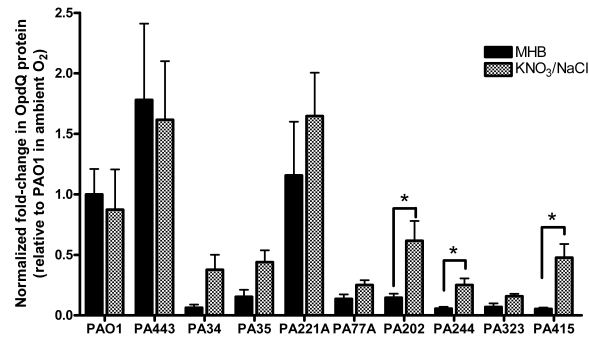

Supplement: Supplementary file 1 — Figure S1. Linear range of detection for Stain‐Free fluorescence of total protein and chemiluminescence signals of the OpdQ protein. (A) Stain‐Free image of the LF‐PVDF membrane containing a dilution series of whole cell lysate from Pseudomonas aeruginosa PAO1. The amount of protein loaded included – lane 1: 80 μg; lane 2: 40 μg; lane 3: 20 μg; lane 4: 10 μg; lane 5: 5 μg; lane 6: 2.5 μg; lane 7: 1.25 μg; lane 8: 0.62 μg. The Stain‐Free fluorescence was proportional to the amount of protein transferred to the LF‐PVDF membrane. Below the Stain‐Free image represents the linear regression analysis used to determine the limits of detection of the Stain‐Free signal. Linearity was achieved across 5–80 μg of total protein per lane. (B) Immunoblot image of OpdQ from the same dilution series of whole lysate from P. aeruginosa PAO1 on the LF‐PVDF membrane in (A). The amount of protein loaded included – lane 1: 80 μg; lane 2: 40 μg; lane 3: 20 μg; lane 4: 10 μg; lane 5: 5 μg; lane 6: 2.5 μg; lane 7: 1.25 μg; lane 8: 0.62 μg. The primary antibody specific for OpdQ was diluted 1:20,000 and the goat anti‐rabbit secondary antibody was diluted 1:50,000. Chemiluminescence signal was achieved and detected using the Supersignal West Femto maximum sensitivity detection solution (Thermo Scientific, Rockford, IL) and the ChemiDoc™ MP imager (Bio‐Rad). Below the immunoblot in the linear regression analysis used to identify the linear range of detection for cheminluminescence signal of the OpdQ protein. Linearity was achieved across 5 to 80 μg of total protein per lane. Data represent the mean SD of the chemiluminescence signals (n = 3). Experiments were performed with three independent immunoblots representing three independent protein lysates extracted from PAO1. Data represent the mean ± standard deviation of the Stain‐Free and chemiluminescence signals (n = 3). Figure S2. Luciferase transcription of clone G with and without KNO3 and NaCl in ambient and 2% oxygen. Luciferase transcripti [file MBO3-4-0967-s001.pdf]
