# Supplementary material for: The OpdQ porin of Pseudomonas aeruginosa is regulated by environmental signals associated with cystic fibrosis including nitrate‐induced regulation involving the NarXL two‐component system
Source: Microbiologyopen. 2015 Oct 12;4(6):967–82. doi: 10.1002/mbo3.305 (PMC4694141; doi:10.1002/mbo3.305)
Supplement: Supplementary file 2 — Table S1. Bacterial strains and plasmids used in this study. [file MBO3-4-0967-s002.docx]

| **Table S1. Bacterial strains and plasmids used in this study** | | |
| --- | --- | --- |
| Strains or Plasmid | Description of relevant genotype or phenotype | Reference |
| ***P. aeruginosa* strains** |  |  |
| PAO1 | Wild-type reference prototroph | K. Poole |
| ∆*narL* | PW7548 – *narL*-C06::ISlacZ/hah | C. Manoil |
| PAO1∆Q | *opdQ::lox* mutant of PAO1 | This study |
| PA443 | Non-mucoid clinical strain from CF patient | Fowler and Hanson (2014) |
| PA34 | Mucoid clinical strain from CF patient | This study |
| PA35 | Non-mucoid clinical strain from CF patient | This study |
| PA221A | Non-mucoid clinical strain from CF patient | This study |
| PA77A | Non-mucoid clinical strain from CF patient | Wolter *et al.* (2008) |
| PA202 | Non-mucoid clinical strain from CF patient | Wolter *et al.* (2008) |
| PA244 | Mucoid clinical strain from CF patient | Wolter *et al.* (2008) |
| PA323 | Mucoid clinical strain from CF patient | Wolter *et al.* (2008) |
| PA415 | Mucoid clinical strain from CF patient | Wolter *et al.* (2008) |
| ***E. coli* strains** |  |  |
| S17-1 | *λpir*; *hsdR pro recA*; RP4 2-Tc::Mu-Km::Tn7, pro, res−, mod+, Str^R^, Trm^R^ | Simon *et al.* (1983) |
| NEB 5α | DH5α^™^ derivative, *fhuA2 ∆ (argF-lacZ) U169 phoA glnV44 ϕ80∆(lacZ)*M15 *gyrA96 recA1 relA1 endA1 thi-1 hsdR17* | NEB |
| **Plasmids** |  |  |
| pMP220 | Broad-host-range, low-copy-number promoter vector, IncP replicon, Tet^R^ Tra | Spaink et al. (1987) |
| pJET1.2 | Blunt cloning vector, Amp^R^ | Thermo Scientific |
| pSPluc+ | Luciferase fusion plasmid, ColE1 replication origin; Amp^R^ | Promega |
| pUCGmlox | Amp^R^, Gm^R^, pUC18-based vector containing the lox flanked *aacC1* gene | Quenee *et al.* (2005) |
| pEX100Tlink | Amp^R^ *sacB*, pUC19-based gene replacement vector with a MCS | Quenee *et al.* (2005) |
| pCM157 | Tet^R^, *cre* expression vector | Marx and Lidstrom (2002) |
| pEX∆QGm | pEX100Tlink containing 5’ and 3’ flanking sequences of *opdQ::Gmlox* | This study |
| p22-*opdQ* | Tet^R^, pMP220 vector containing the 1821 bp upstream fragment of the promoter and entire *opdQ* gene from PAO1 | This study |
| p22*luc* | Tet^R^, pMP220 vector containing the promoterless *luc* gene at the *Kpn*I and *Hind*III sites | This study |
| p22*luc*-KpnOpdQ173 | Tet^R^, p22*luc* plasmid with the 172 bp *opdQ* upstream fragment at *Kpn*I and *Xba*I sites; clone A | This study |
| p22*luc*-KpnOpdQ144 | Tet^R^, p22*luc* plasmid with the 143 bp *opdQ* upstream fragment at *Kpn*I and *Xba*I sites; clone B | This study |
| p22*luc*-KpnOpdQ149 | Tet^R^, p22*luc* plasmid with the 137 bp *opdQ* upstream fragment at *Kpn*I and *Xba*I sites; clone C | This study |
| p22*luc*-KpnOpdQ109 | Tet^R^, p22*luc* plasmid with the 109 bp *opdQ* upstream fragment at *Kpn*I and *Xba*I sites; clone D | This study |
| p22*luc*-KpnOpdQ97 | Tet^R^, p22*luc* plasmid with the 96 bp *opdQ* upstream fragment at *Kpn*I and *Xba*I sites; clone E | This study |
| p22*luc*-KpnOpdQ78 | Tet^R^, p22*luc* plasmid with the 77 bp *opdQ* upstream fragment at *Kpn*I and *Xba*I sites; clone F | This study |
| p22*luc*-KpnOpdQ23 | Tet^R^, p22*luc* plasmid with the 22 bp *opdQ* upstream fragment at *Kpn*I and *Xba*I sites; clone G | This study |
| ^a^Abbreviations: Amp^R^, ampicillin resistance; Tet^R^, tetracycline resistance; Gm^R^, gentamicin resistance; MCS, multiple cloning site. | | |
