# Supplementary material for: The OpdQ porin of Pseudomonas aeruginosa is regulated by environmental signals associated with cystic fibrosis including nitrate‐induced regulation involving the NarXL two‐component system
Source: Microbiologyopen. 2015 Oct 12;4(6):967–82. doi: 10.1002/mbo3.305 (PMC4694141; doi:10.1002/mbo3.305)
Supplement: Supplementary file 3 — Table S2. Primers used in this study. [file MBO3-4-0967-s003.docx]

| Table S2. Primers used in this study | | | | |
| --- | --- | --- | --- | --- |
| Primer^a^ | **Sequence (5’- 3’)^b^** | **PCR Product Size (bp)** | **Annealing Temperature^d^** | **Application^e^** |
| opdQ5’RACE1 | ccgatcttcagggtgctcttcg | -^c^ | 55°C | 5’RACE |
| opdQ5’RACE2 | ATCCCTGCCTCGCCATAGTCG |  | 58°C |  |
| LucRTF | CTCACTGAGACTACATCAGC | 160 | 55°C | Expression |
| LucRTR | GACCTCTCACACACAGTTC |  |  |  |
| OpdQRTF | CCTGAAGAAGGTCAACCAGCG | 158 | 55°C | Expression |
| OpdQRTR | CGAACTTGTCCGAAGTCAGCC |  |  |  |
| RpoDRTF | gagctgttcatgccgatcaagc | 211 | 55°C | Expression |
| RpoDRTR | GCTTGCTCTTCAGGACGCTGTCG |  |  |  |
| KpnOpdQ173F | **GGTACC**AATAGCGGGAAAGC | 172 | 69°C | Clone A |
| KpnOpdQ144F | **GGTACC**TTCGTGCCAATTCGCGGCC | 143 | 69°C | Clone B |
| KpnOpdQ149F | **GGTACC**AATAGCGGGAAAGCCCGTCCTAGACAGGGTTCGTGCCAATTCGCGGCCACCCTCGACTAATGTCTAACAGTGGCCGTCTTCCTGTAGTGCTTAAGTAACCGGGCTGTCACGCCCCAAGCGCTCGAGGAGACAATAAC**AAGCTT** | 137 | 69°C |  |
|  |  |  |  |  |
|  |  |  |  | Clone C |
|  |  |  |  |  |
|  |  |  |  |  |
|  |  |  |  |  |
| KpnOpdQ109F | **GGTACC**taacagtggccgtcttcc | 109 | 69°C | Clone D |
| KpnOpdQ97F | **GGTACC**TCTTCCTGTAGTGC | 96 | 69°C | Clone E |
| KpnOpdQ78F | **GGTACC**TAACCGGGCTGTCACAGG | 77 | 69°C | Clone F |
| KpnOpdQ23F | **GGTACC**AAGCGCTCGAGG | 22 | 69°C | Clone G |
| HindOpdQRBS | CG**AAGCTT**GTTATTGTCTCCTCG | -^c^ | 69°C | Clones A-G |
| XbaLucR1 | C**TCTAGA**ATTACACGGCGATCTTTCC |  |  |  |
| opdQ5’EcoF | GCT**GAATTC**CTAGACAGGGTTCGTGC | 716 | 58°C | Knockout |
| opdQ5’HindR | CGT**AAGCTT**GGTTGACCTTCTTCAGGC |  |  |  |
| opdQ3’HindF | GCA**AAGCTT**ACAACCTCAGCACCAGC | 881 | 58°C | Knockout |
| opdQ3’BamR | GTAT**GGATCC**GCATCCGCTTGAGC |  |  |  |
| aacCF1 | GCTTGGTGCTTATGTGATCTACG | - ^c^ | 55-58°C | Knockout |

^a^Primers incorporated either a *Kpn*I, *EcoR*I, *BamH*I, or *Hind*III restriction site indicated by Kpn, Hind, or Bam, except for clone KpnOpdQ149F which contained both *Kpn*I and *Hind*III sites in the oligonulceotide.

^b^Underlined and bold sequences represent incorporated restriction sites in the primer sequence.

^c^Not applicable, PCR product sizes varied. HindOpdQRBS was the reverse primer used to generate the initial full-length promoter that was fused to luciferase, whereas XbaLucR1 was the reverse primer used to amplify the promoter::luciferase fragments that were cloned into pMP220.

^d^PCRs were conducted with regular *Taq* polymerase or the Phusion Hot Start II polymerase with a final concentration of 2 mM MgCl_2_.
